# Navigating Challenges and Treatment Options in Diaphyseal Forearm Fractures Among Adolescents: Case Series and Narrative Review

**DOI:** 10.7759/cureus.40669

**Published:** 2023-06-19

**Authors:** Takaki Yoshiyama

**Affiliations:** 1 Orthopaedics, Osaka Red Cross Hospital, Osaka, JPN

**Keywords:** hybrid method, double plate, intramedullary nails, re-fractures, fractures in children, forearm diaphyseal fractures

## Abstract

In this paper, we recount the medical trajectories of two male patients, both fourteen years of age, who sustained re-fractures of their radius and ulna six months post their primary diaphyseal fractures. Owing to the limited capacity for growth of the forearm bones between the ages of ten to sixteen years, many queries are engendered concerning apt treatment strategies. The pressing questions are whether these should be conservative or surgical and the precise method to be employed in surgical interventions. This discourse endeavors to demarcate preferred therapeutic options and shed light on a series of standard clinical dilemmas physicians encounter, along with an exhaustive scrutiny of existing literature.

## Introduction

Forearm diaphyseal fractures are prevalent during childhood, constituting 17.8%-26% of all fractures [1,2]. Among these, concurrent fractures of both forearm bones account for 3.4-5.4% of all fractures and are commonly encountered by orthopedic surgeons [2,3]. While a favorable functional prognosis is reported in over 90% of such fractures [2], children older than 10 years have limited growth potential, which reduces remodeling potential and complicates conservative or surgical management. We present two cases of 14-year-old boys who experienced re-fracture more than six months post-initial fracture, necessitating revision surgery. We review the treatment course and provide a literature review of clinical queries to assist physicians in managing similar cases in the future.

This paper elucidates the optimal treatment of diaphyseal forearm fractures in early adolescents, synthesizing current knowledge and offering recommendations for ideal management. Through a case series, we contribute to the ongoing discourse and refinement of treatment approaches for this susceptible population.

## Case presentation

Case 1

**Figure 1:**
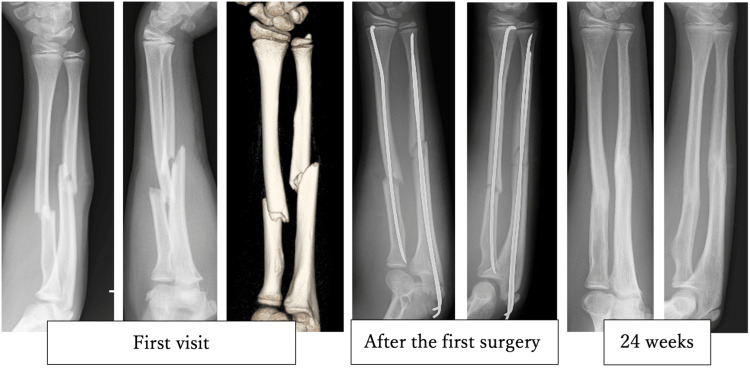
The initial adolescent male, aged 14 X-rays for the first visit, after the first surgery and 24 weeks. The patient suffered fractures in both the radius and ulna bones of the forearm, which were then surgically treated, with a Kirschner wire (K-wire) utilized for stabilization. This wire was removed 14 weeks post-surgery, and by the 24th week, the bones had fused.

**Figure 2:**
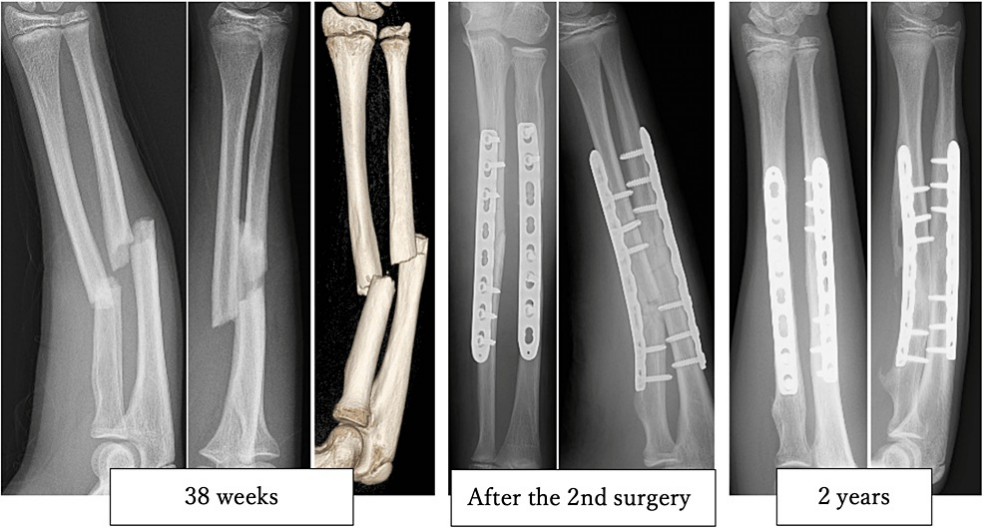
Subsequent image of the aforementioned 14-year-old male X-rays taken at 38 weeks, and at 2 years, revealed that the radius and ulna had re-fractured; the bones were then double-plated for stabilization within the same week. The bones fused properly post-operation and remained stable for 2 years afterward.

A 14-year-old boy fell while playing soccer and forcefully struck his hands on the ground. He subsequently visited our hospital with a chief complaint of left forearm deformity. Diaphyseal fractures of both left forearm bones were identified, and emergency surgery was performed the same day to insert Kirschner wire (K-wire) into the radius and ulna as intramedullary fixation (Figure 1). The radius needed to be mini-opened for reduction. Postoperatively, the patient was immobilized externally for seven weeks, and the K-wire was removed at 14 weeks. At this time, the range of motion of the elbow joint was 0-140 degrees, with 90 degrees of pronation and supination, scoring excellent on the Grace-Eversmann functional assessment (Table 1). After resuming sports at 24 weeks, the patient experienced a re-fracture at 38 weeks, necessitating a second surgery involving open reduction and internal fixation of both the radius and ulna (Figure 2). The surgery was challenging due to tissue adhesion during deployment using Henry's approach, partly attributable to the initial mini-opening procedure. The patient is scheduled to have the plates removed in approximately 2.5 years. At the last follow-up (two years post-initial surgery), the range of motion of the elbow joint was 0-140 degrees, with 90 degrees of pronation and supination, and an excellent Grace-Eversmann functional assessment score.

**Table 1:** Grace and Eversmann functional evaluation criteria

	Union	Pronation supination comparison ratio with the uninjured arm
Excellent	+	90-100%
Good	+	80-89%
Acceptable	+	60-79%
Unacceptable	−	<60%

Case 2

**Figure 3:**
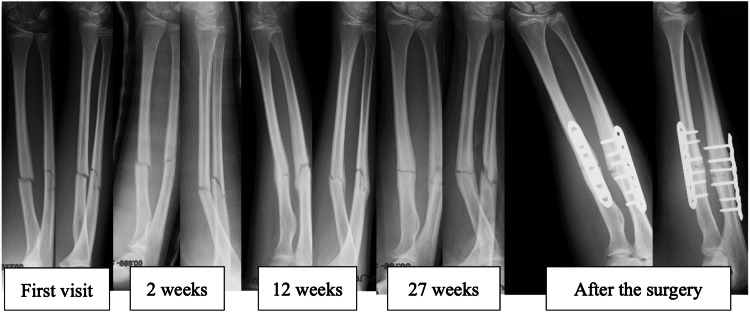
The secondary 14-year-old male X-ray observations were made at the initial visit, 2 weeks, 12 weeks, 27 weeks, and post-surgery in the same week. The minor dislocation was noted initially, with a slight angular deformity developing by the 2-week mark. No further deformity progression was observed, and bone fusion was achieved at 12 weeks. However, a re-fracture occurred at 27 weeks, leading to the performance of a double-plate procedure.

A 14-year-old male patient with no notable medical history presented to our hospital after incurring diaphyseal fractures of the forearm due to a forceful impact on the ground during a fall. Initial examination revealed closed fractures with no neuropathic indications, accompanied by mild swelling. Because of the presence of a minimal angular deformity, a conservative treatment approach was adopted. A follow-up radiographic examination at two weeks detected a 15-degree angulation in the radius, which, considering the patient's overall condition, was deemed acceptable. Therefore, the conservative treatment, involving cast immobilization of the upper arm, continued. Following eight weeks of cast immobilization, the patient was permitted to engage in exercises involving the flexion and extension of the elbow, and the supination and pronation of the wrist joint. A slight stiffness in supination was reported during this period. Radiographic confirmation of successful bony fusion was obtained at the 12-week mark, and the patient scored 'excellent' on the Grace-Eversmann functional assessment due to an elbow joint range of motion between 0-140 degrees, alongside 90 degrees of internal and external rotation. Regrettably, 27 weeks after the initial fracture, the patient experienced a re-fracture in the same region due to another fall. Consequently, an open surgical procedure was performed on both the radius and ulna two days subsequent to the patient's visit (Figure 3).

## Discussion

Diaphyseal forearm fractures within this demographic prove challenging to alleviate, as re-fractures may transpire even after successful fusion. Males within this age bracket frequently engage in athletic activities and may exhibit suboptimal adherence to medical recommendations. This study examines the literature to address key clinical questions in managing such cases.

Initially, we shall deliberate on whether the re-fracture incidence diverges between conservative and surgical interventions. Sinikumpu et al. conducted a comparative analysis, finding a 7.1% re-fracture prevalence, with no significant difference between the two cohorts, and a 1.2% nonunion rate below the age of 16 [4]. The review ascertained that high-energy trauma and chronic ailments exacerbate nonunion risks. The extent of permissible angulation and malrotation, from a conservative standpoint, is contingent upon the patient's age; the criteria delineated by Noonan and Price in 1998 (Table 2) garner support from multiple contemporary authors and appear to function as a valuable benchmark [5-8].

**Table 2:** Noonan and Price Criteria

Patient age	Angulation	Rotation	Bayonet apposition
Age 0–9 (0–8 girls, 0–10 boys)	<15°	<45°	Up to 1 cm
Age >9 (>8 girls, >10 boys)	<10° proximal/midshaft	<30°	Up to 1 cm
Age >9 (>8 girls, >10 boys)	<15° distal	<30°	Up to 1 cm

Subsequently, we explore surgical intervention options, comparing intramedullary nails and plates. While implementing an intramedullary fixation with a K-wire, Dietz et al. found that positioning the K-wire in both bones is advantageous, as fixation applied only to the ulna for diaphyseal fractures resulted in persistent angulation in the radius for 29% of cases [9]. A systematic review identified no notable disparities in functional outcomes, fracture union duration, complication rates, bone angulation, or rotation between plates and intramedullary nails [3]; however, this report remains somewhat contentious. Freese et al. disclosed that radiographic fusion duration was significantly prolonged in patients undergoing intramedullary fixation compared to those treated with plates, with 68 days versus 58 days [10]. The author deems the findings of this report more credible, acknowledging that those familiar with Arbeitsgemeinschaft Osteosynthese Fragen (AO) principles can readily comprehend that in locking plate fixation cases, "absolute stability" is achieved, precluding micromotion. Conversely, intramedullary nail fixation employs "relative stability," permitting micromotion and promoting secondary fusion through callus formation. It is more plausible to presume that the latter necessitates marginally extended healing time; thus, postoperative monitoring should be executed with the understanding that fusion duration will be slightly protracted when selecting an intramedullary nail over a plate in children aged 10-16 years.

Next, we discuss plate fixation techniques and the possibility of bone atrophy due to stress shielding. Matsuura et al. postulated that a conventional plate might be less likely to provoke bone atrophy compared to a locking plate [11]. Nonetheless, the decrease in bone density reportedly commences in the fourth to fifth year [12,13], which may not be problematic for this age group as implants are removed within approximately two or three years. Nonetheless, this factor may warrant consideration in adults who retain their implants.

Following, the author will examine a surgical technique employing both a plate and an intramedullary fixation, referred to as the hybrid method. Plate fixation in children fundamentally assumes that the plate will eventually be removed. Plate fixation to the ulnar diaphysis is relatively straightforward; however, when utilizing Henry's approach to the radial diaphysis, numerous anatomical structures warrant preservation, such as the radial artery, radial nerve, and posterior interosseous nerve, potentially causing discomfort for many surgeons [14]. This apprehension would be exacerbated during removal surgery due to tissue adhesions. In this context, the hybrid approach of plate fixation solely on the ulna and percutaneous K-wire intramedullary nailing on the radius appears to be a compelling treatment alternative, provided the outcome is favorable.

The hybrid technique has been documented in numerous publications, boasting favorable outcomes [15-18]. Zheng et al. evaluated the results of 137 patients, aged 10-16 years, who underwent surgery for bilateral forearm diaphyseal fractures, comparing the double plate, double intramedullary nail, and hybrid groups [16]. The hybrid group demonstrated significantly reduced operative duration, incision length, and intraoperative blood loss compared to the double plate group. Furthermore, the authors reported the hybrid group's advantages of shorter intraoperative fluoroscopy time and reduced postoperative fixation duration relative to the double intramedullary nail group. Moreover, the ulnar union rate at three months postoperatively was significantly lower in the double intramedullary nail group compared to both the hybrid and double plate groups; however, the hybrid group exhibited comparable results to the double plate group. Consequently, the hybrid approach would be a valuable technique.

In light of these findings, the flowchart presented in Caruso et al.'s report [8] proves to be exemplary, substantiated, and beneficial for determining appropriate treatment strategies.

Lastly, we consider the appropriate timing for resuming sports and other activities following the removal surgery. Adolescents within this age group frequently participate in sports and may struggle with adherence, potentially disregarding medical guidance such as activity restrictions. As per the study by Matsuura et al. previously mentioned, bone strength was assessed preoperatively, as well as one, three, and six months post-plate removal [13]. Although bone strength exhibited near-complete recovery six months after removal, certain regions, such as the distal portion of the ulna, demonstrated insufficient recuperation.

It has been posited that implementing protective bracing and a six-month athletic restriction may diminish the re-fracture rate in patients aged 6-11 years [19]; thus, imposing a six-month sports ban following plate removal could be a prudent measure. Heightened caution is warranted in cases of overweight or obese children. Compromised outcomes in forearm fractures have been documented in obese patients, encompassing an elevated risk of fracture or injury [20,21], unsuccessful closed reduction [22], and loss of reduction during conservative management [23,24]. In the case of children with obesity, it might be advisable to reduce the surgical threshold and extend the duration before resuming sports activities beyond the standard time frame.

## Conclusions

Re-fracture rates do not differ significantly between conservative and surgical treatments. Intramedullary nailing, plate fixation, and hybrid methods are viable surgical options. Hybrid methods show favorable outcomes, with shorter operative time and reduced blood loss. Post-surgical care, including sports restrictions and bracing, is crucial in preventing re-fractures.
